# Examining Topoisomers of a Snake-Venom-Derived Peptide for Improved Antimicrobial and Antitumoral Properties

**DOI:** 10.3390/biomedicines10092110

**Published:** 2022-08-29

**Authors:** Adam Carrera-Aubesart, Sira Defaus, Clara Pérez-Peinado, Daniel Sandín, Marc Torrent, Maria Ángeles Jiménez, David Andreu

**Affiliations:** 1 Proteomics and Protein Chemistry Unit, Department of Medicine and Life Sciences, Pompeu Fabra University, 08003 Barcelona, Spain; 2 Systems Biology of Infection Lab, Department of Biochemistry and Molecular Biology, Facultat de Biociències, Universitat Autònoma de Barcelona, 08193 Bellaterra, Spain; 3 Institute of Physical Chemistry “Rocasolano” (IQFR), Consejo Superior de Investigaciones Científicas (CSIC), 28006 Madrid, Spain

**Keywords:** antimicrobial peptides, snake venom, crotalicidin, Ctn[15-34], enantio-, retro-, and retroenantio peptides, topoisomer peptides, antitumoral peptides

## Abstract

Ctn[15-34], the C-terminal section of crotalicidin (Ctn), a cathelicidin from a South American pit viper, is an antimicrobial and antitumoral peptide with remarkably longer stability in human serum than the parent Ctn. In this work, a set of topoisomers of both Ctn and Ctn[15-34], including the retro, enantio, and retroenantio versions, were synthesized and tested to investigate the structural requirements for activity. All topoisomers were as active as the cognate sequences against Gram-negative bacteria and tumor cells while slightly more toxic towards normal cells. More importantly, the enhanced serum stability of the D-amino-acid-containing versions suggests that such topoisomers must be preferentially considered as future antimicrobial and anticancer peptide leads.

## 1. Introduction

Since the discovery of penicillin by Fleming in 1928 [1] and the ensuing golden era of antibiotic development (1930–1960), the prospects for anti-infective therapies have become increasingly problematic due to the rise in antimicrobial resistance (AMR), first observed as the ability of *E. coli* to synthesize penicillinase that inactivated penicillin [2], later (1960) as the lack of response of 80% of *S. aureus*-infected patients to penicillin [3], and since then on to the current, widespread AMR crisis, an alarming predicament that is unanimously regarded as one of the most serious threats to global health [4,5,6,7].

In order to fight AMR, it is essential to have a good understanding of the mechanisms underlying infection, in particular those related to the interaction between anti-infective agents and pathogen targets. In this regard, antimicrobial peptides (AMPs) are emerging as promising therapeutic alternatives in the worrying context of global AMR for reasons that include, among others, their simple but effective mechanisms of action—different from traditional antibiotics and with a low likelihood of giving rise to resistance [8]—broad spectrum (Gram-negative and -positive bacteria, fungi, protozoa, and viruses [9,10,11]), amenability to structural modification by versatile peptide engineering techniques, and affordable production costs. In addition, AMPs are also showing promise as potential antitumor agents [12]. Structurally, AMPs comprise a wide variety of motifs; although they share a generally cationic nature and a high content of hydrophobic residues, both features combine to promote amphipathic secondary structures that facilitate membrane interaction and ensuing biological action [13,14].

The cathelicidins are one of the main families of AMPs, widely distributed through the animal kingdom (from human LL-37 [15] to primitive hagfish species [16]), with an important role in host defense based on their broad antimicrobial activity [17]. The name cathelicidin refers to a primary structure where a conserved N-terminus harboring a signal peptide and a cathepsin L inhibitor (cathelin) domain precedes a C-terminal region from which the mature cathelicidin is proteolytically cleaved. Pertinent to this work, cathelicidin-related antimicrobial peptides (CRAMPs) with remarkable antimicrobial activity (Gram-negative bacteria) have been found in the venoms of both Asian and South American snakes [9]. Our laboratory has focused on one such CRAMP, crotalicidin (Ctn) from *Crotalus durissus terrificus* [18], from which a C-terminal fragment results from structure-based rational design, Ctn[15-34] [9,19,20], has been shown to have antimicrobial and antitumor properties similar or superior to the parent Ctn, including remarkable proteolytic stability in biological fluids [9,18].

In peptide research, the topoisomer (the term topoisomer is used to denote composition-identical (hence *isomer*) but three-dimensionally distinct (hence *topo*) variants of a given molecule, with structural changes translating into different bioactivity. Such a broad definition inevitably encompasses a wide variety of structural arrangements of identical molecular composition; in practice, the term is largely confined to the nucleic acid [21,22] and peptide/protein fields [23,24]) label is often used to describe the different spatial patterns multiple disulfide peptides can fold into as a result of different cysteine pairings. Less frequent but equally appropriate use of the term concerns peptide structures with altered sequence and/or stereochemical features within a fixed composition. This still rather broad category may be further narrowed down to peptides such as those in this work, i.e., with fully inverted amino acid sequence (retro isomers) or with the canonic sequence made up of residues of reversed chirality (enantio isomers), or with both features combined (retroenantio isomers). Research in this area over recent years has unveiled rather interesting features of these isomers, particularly the retroenantio ones [23,24,25,26,27].

Against this backdrop, the present study focuses on the topoisomers that result from applying retro, enantio, and retroenantio modifications to the original Ctn and Ctn[15-34] sequences. We hence report the synthesis of the six peptides (three each for Ctn and Ctn[15-34], Table 1), comment on their structural features, in particular, the similarities found by solution nuclear magnetic resonance (NMR) between the cognate and retroenantio versions, and examine their in vitro activity on both antimicrobial and antitumor screens, as well as their stability in human serum.

## 2. Materials and Methods

### 2.1. Peptide Synthesis

Ctn (KRFKKFFKKVKKSVKKRLKKIFKKPMVIGVTIPF), Ctn[15-34] (KKRLKKIFKKPMVIGVTIPF), and their six topoisomers (Table 1) were synthesized in C-terminal carboxamide form in a Liberty Blue instrument (CEM Corporation, Matthews, NC, USA) using Fmoc solid phase peptide synthesis (SPPS) at 0.1 or 0.25 mmol scale on Rink Amide ProTide resin. Side chain functionalities were protected with *tert*-butyl (Ser, Thr), *tert*-butyloxycarbonyl (Lys), and 2,2,4,6,7-pentamethyldihydrobenzo- furan-5-sulfonyl (Arg) groups. Four equivalents of Fmoc-L- or Fmoc-D-amino acid derivative and equivalent amounts of Oxyma Pure (Merck, Darmstadt, Germany) and *N*,*N′*-diisopropylcarbodiimide were used for the couplings, with *N*,*N*-dimethylformamide (DMF) as solvent. After chain assembly, full deprotection and cleavage from the resin were conducted with trifluoroacetic acid (TFA)/H_2_O/triisopropylsilane (95:2.5:2.5 *v*/*v*, 120 min, rt.). Peptides were precipitated with cold diethyl ether, dissolved in water, and lyophilized.

### 2.2. Peptide Analysis

Analytical reversed-phase HPLC or UHPLC were performed on C18 columns (4.6 × 50 mm, 3 μm; Phenomenex) in LC2010A or LC2040C3DPlus instruments (Shimadzu Corp., Kyoto, Japan). Linear gradients (15–50%) of solvent B (0.036% TFA in MeCN) into A (0.045% TFA in H_2_O) were used for elution at 1 mL/min (HPLC) or 0.6 mL/min (UHPLC) flow rate, with UV detection at 220 nm. LC-mass spectrometry was performed in an LC-MS 2010EV instrument (Shimadzu, Kyoto, Japan) fitted with an XBridge column (4.6 × 150 mm, 3.5 μm; Waters, Cerdanyola del Vallès, Spain), eluting with a 15–50% linear gradient of B (HCOOH/MeCN 0.08% *v*/*v*) into A (HCOOH/H_2_O 0.1%, *v*/*v*) over 15 min at 1 mL/min with UV detection at 220 nm.

### 2.3. Peptide Purification

Preparative HPLC runs were performed on a Luna C18 column (21.2 mm × 250 mm, 10 μm; Phenomenex), using linear gradients (15–50%) of solvent B (0.1% TFA in MeCN) into A (0.1% TFA in H_2_O), as required, at a flow rate of 25 mL/min. Fractions with adequate HPLC homogeneity (>95%) and the expected mass by LC-MS were pooled, lyophilized, and used in subsequent experiments. All the peptides were solved in water for resuspension.

### 2.4. NMR Spectroscopy

Samples were prepared by dissolving the lyophilized peptide (1–2 mg) in 0.5 mL of a fresh solution of 30 mM DPC-d38 (98% deuteration; Cambridge Isotope Laboratories, Inc Andover, MA) at pH 3.0 in either H_2_O/D_2_O (9:1 *v*/*v*) or pure D_2_O. Peptide concentrations were approximately 1 mM. Sodium 2,2-dimethyl-2-silapentane-5-sulfonate was added as an internal reference. pH was measured with a glass microelectrode (not corrected for isotope effects) and adjusted, if necessary, by adding minimal amounts of NaOD or DCl.

NMR spectra were recorded in a Bruker AVNEO-600 spectrometer operating at a 600.13 MHz proton frequency and equipped with a cryoprobe. Probe temperature was calibrated using a methanol sample; 1D and 2D spectra, i.e., double-filtered-quantum-phase-sensitive correlated spectroscopy (DFQ-COSY), total correlated spectroscopy (TOCSY), nuclear Overhauser enhancement spectroscopy (NOESY), and ^1^H−^13^C heteronuclear single quantum coherence spectra (HSQC), were acquired using standard pulse sequences, and processed with the TOPSPIN program (Bruker Biospin, Karlsruhe, Germany), as reported [28]. TOCSY and NOESY mixing times were 60 and 150 ms, respectively. The water signal was suppressed using an excitation sculping scheme [29]. ^1^H−^13^C HSQC spectra were acquired at natural heteronuclear abundance. The ^13^C δ-values were indirectly referenced using the IUPAC-recommended ^13^C/^1^H ratio of 0.25144953 [30].

^1^H and ^13^C assignment was achieved by standard sequential analysis [31,32] of 2D DFQ-COSY, TOCSY, and NOESY spectra acquired at 25 °C and 35 °C, examined in combination with the corresponding 2D ^1^H−^13^C HSQC spectra. The ^13^C chemical shift values served to confirm the assignment of side chains, in particular the repeated Lys residues. The spectra were analyzed using the NMRFAM-SPARKY software [33]. The assigned chemical shifts have been deposited at the BioMagResBank (http://www.bmrb.wisc.edu (accessed on 28 July 2022)) with accession codes 51536 (Ctn retroenantio) and 52537 (Ctn[15-34] retroenantio).

Secondary structure was delineated from the ^1^Hα and ^13^Cα chemical shift deviations, which are defined as Δδ_Hα_ = δ_Hα_^observed^ − δ_Hα_^RC^, ppm, and Δδ_Cα_ = δ_Cα_^observed^ − δ_Cα_^RC^, ppm, being δ_Hα_^observed^ and δ_Cα_^observed^ the ^1^Hα and ^13^Cα chemical shifts presented for the peptides, and δ_Hα_^RC^ and δ_Cα_^RC^ the random coil values, which were taken from Wishart et al. [34]. Negative Δδ_Hα_ and positive Δδ_Cα_ values are indicators of helical structures, and positive Δδ_Hα_ and negative Δδ_Cα_ values of β-sheets/extended structures are considered as random coil or disordered those with small absolute values (approximately |Δδ_Hα_| ≤ 0.04 ppm and |Δδ_Cα_| ≤ 0.4 ppm).

The percentages of helical populations were estimated from the Δδ_Hα_ values averaged for all the helical residues (Δδ_Hα_^Av^, ppm) by applying Equation (1), as previously described [35,36].

% helix = 100 × (Δδ_Hα_^Av^/Δδ_Hα_^100%helix^) (1)

The Δδ_Hα_ value for 100% helix (Δδ_Hα_^100%helix^) was taken as −0.39 ppm, which is the averaged value in protein helices.

### 2.5. NMR Structure Calculation

Structure calculations were performed with the CYANA 3.98 program [37,38]. Upper limit distance restraints were obtained from the NOE cross-peaks present in 2D NOESY spectra recorded at 25 °C and 35 °C, which were integrated using the automatic integration subroutine of the Sparky program (T. D. Goddard and D. G. Kneller, Sparky 3, NMR assignment program, University of California, San Francisco, CA, USA). In addition, restrictions for i, i + 4 hydrogen bonds were incorporated for the helical regions, as deduced from the δ_Hα_ and δ_Cα_ values (Figures S1 and S2). The standard iterative protocol for automatic NOE assignment implemented in CYANA 3.98 was used. It consists of seven cycles of combined NOE assignment and structure calculation of 100 conformers per cycle. For each peptide, the final NMR structure corresponds to the ensemble of the 20 conformers with the lowest target function value. MOLMOL [39] was used to visualize the structures of the peptides. Structures are available upon request from the authors.

### 2.6. Bacterial Strains and MIC

Assays were performed on three Gram-negative bacteria: *E. coli* (BW25113, Coli Genetic Stock Center), *A. baumannii* (ATCC 15308, CECT, Valencia, Spain), and *Pseudomonas* sp. (ATCC15915, CECT). The minimal inhibitory concentration (MIC) of each peptide was determined as described by Wiegand et al. [40]. Peptide stocks in water were added to polypropylene 96-well plates (Greiner, Frickenhausen, Germany) and serially diluted from 100 to 0.2 μM. Fresh bacteria were incubated at 37 °C in Mueller Hinton Broth (MHB, Condalab, Torrejón de Ardoz, Spain) up to exponential growth and diluted to a final inoculum of 5 × 10^5^ CFU/mL. BSA and acetic acid at 0.04% (*w*/*v*) and 0.002% (*v*/*v*) final concentration, respectively, were added to avoid peptide self-aggregation. Plates were incubated for 20–22 h at 37 °C. Each peptide was tested in duplicate. MIC values were the lowest peptide concentration where bacterial growth was not detected.

### 2.7. Cell Culture

Human HeLa S3 (cervix epithelial carcinoma), leukemia Jurkat E6.1 (T-cell lineage), U937 (histiocytic lymphoma), THP-1 (acute monocytic leukemia), and 1BR3G human fibroblasts were obtained from the Cell Lines Repository of the Institut Municipal d’Investigació Medica (Barcelona, Spain). BxPC3 (pancreas adenocarcinoma), A549 (lung carcinoma), SH-Sy5Y (neuroblastoma), Panc1 (epithelioid carcinoma), 786-O (renal cell adenocarcinoma), and HT29 (colorectal adenocarcinoma) were obtained from the Integrative Biomedical Materials and Nanomedicine lab at UPF (Barcelona, Spain). HeLa S3, Jurkat E6.1, U937, THP-1, Panc1, and 786-O were cultured in Roswell Park Memorial Institute medium (RPMI) supplemented with 10% fetal bovine serum (FBS) and 1% penicillin/streptomycin solution and maintained at 37 °C in a humidified atmosphere with 5% of CO_2_. 1BR3G, BxPC3, A549, and HT29 were cultured in Dulbecco’s Modified Eagle’s Medium (DMEM) supplemented with 10% FBS and 1% penicillin/streptomycin solution and maintained at 37 °C in a humidified atmosphere with 5% of CO_2_. SH-Sy5Y was cultured in DMEM supplemented with 15% FBS and 1% penicillin/streptomycin and maintained at 37 °C in a humidified atmosphere with 5% of CO_2_. Cultures were maintained at 10^5^–10^6^ cell/mL densities. For 1BR3G human fibroblasts, BxPC3, A549, SH-Sy5Y, Panc1, 786-O, and HT29 were split every time they reached 80–90% of confluence after harvesting with PBS and trypsin.

### 2.8. Peptide Cytotoxicity

About 60,000 cells were added to different microfuge tubes containing 2-fold serial dilution of the peptide, with a final concentration in the range between 0.1–100 μM in RPMI containing 2% of FBS. After 30 min incubation at 37 °C and 5% of CO_2_, 100 mL of medium containing approximately 10,000 treated cells were transferred to a 96-well plate. Then, 15 μL of Cell Titer Blue dye (Promega, Madison, WI, USA) was added, and plates were reincubated for 24 h. Fluorescence was read at 4 and 24 h after dye addition in a Synergy HTX (BioTek, Winooski, VT, USA) reader, with λ_exc_ = 560 nm and λ_em_ = 620 nm. In the case of adherent cultures, 5000 cells/well were seeded in 96-well plates. After 24 h incubation at 37 °C and 5% of CO_2_, the medium was removed, and a new medium with 2% of FBS containing the various 2-fold dilution of the peptides was added. After a further 30 min, 15 μL of Cell Titer Blue dye was added to each well, and the measurement was done as above. Relative viability was calculated relative to a cell with only 2% FBS in the corresponding medium as a control (100% viability). All assays were conducted in triplicate.

### 2.9. Hemolytic Activity

Fresh human blood (10 mL) was collected in EDTA tubes and centrifuged at 1000× *g* for 10 min at 4 °C. After plasma removal, the pellet containing the erythrocytes was washed 3× with phosphate-buffered saline (PBS) and resuspended to an 8% (*v*/*v*) suspension in PBS. Then, 100 μL of the suspension was added to a final concentration of 4% (*v*/*v*) to microfuge tubs containing 2-fold serially diluted peptides (0.2–100 μM). The suspension was incubated for 30 min at 37 °C with agitation, then centrifuged for 2 min at 1000× *g*. The supernatant was transferred to 96-well plates, and hemoglobin was measured by OD at 540 nm in a Synergy HTX plate reader (BioTek). Positive and negative controls were 1% (*v*/*v*) Triton X-100 and untreated erythrocytes (4% *v*/*v* in PBS), respectively. Measurements were done in triplicate. Hemolysis was calculated as:

(2) $$\%\;Hemolysis=\frac{\left[OD_{540}^{peptide}\right]-\left[OD_{540}^{PBS}\right]}{\left[OD_{540}^{Triton}\right]-\left[OD_{540}^{PBS}\right]}\cdot 100$$

### 2.10. Serum Stability

Briefly, 0.5 mL each of human serum (Sigma-Aldrich, St. Louis, MO, USA) and peptide (1 mM in water) were mixed and incubated for 24 h at 37 °C with stirring. Aliquots (0.1 mL) were taken at 0, 1, 5, 10, 30, 120, 360 and 1440 min and treated with 20 μL of trichloroacetic acid (15% (*v*/*v*) in water). The suspension was centrifuged for 30 min at 4 °C and 13,000 rpm, and the supernatant was analyzed by HPLC as described above.

## 3. Results

### 3.1. Synthesis of Ctn and Ctn[15-34] Topoisomers

Since the Ctn and Ctn[15-34] sequences have proven to be valuable antimicrobial (AMP) and antitumor peptide (ATP) leads, we set out to decipher if their topoisomers have similar or even improved properties [9]. Table 1 shows a set of C-terminally amidated topoisomers produced by standard Fmoc solid-phase peptide synthesis (SPPS) methods. The analytical characterization is in the supplementary information (Table S4, pages S12–S14).

### 3.2. Minimal Inhibitory Concentration (MIC)

Ctn, Ctn[15-34], and their topoisomers were tested in minimal inhibitory concentration (MIC) assays against three representative Gram-negative bacteria (*Pseudomonas* sp., *Escherichia coli,* and *Acinetobacter baumannii*) (Table 2). The topoisomers were not tested against Gram-positive bacteria, as previous results for the cognate Ctn and Ctn[15-34] showed preferential activity against Gram-negative [9]. Comparing MIC values, we observed that while Ctn topoisomers lost around 50% activity in comparison to their precursor, Ctn[15-34] topoisomers performed almost undistinguishably from the original, thus qualifying as good AMP leads.

### 3.3. Cell Viability Studies

**Figure 1:**
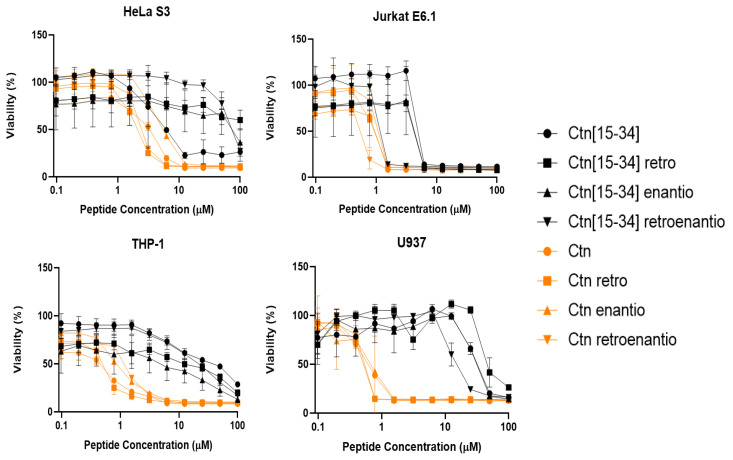
Viability of leukemia cells upon peptide treatment for 24 h. Ctn[15-34] and Ctn topoisomers are depicted in black and orange, respectively. Results are the average of three replicates.

**Figure 2:**
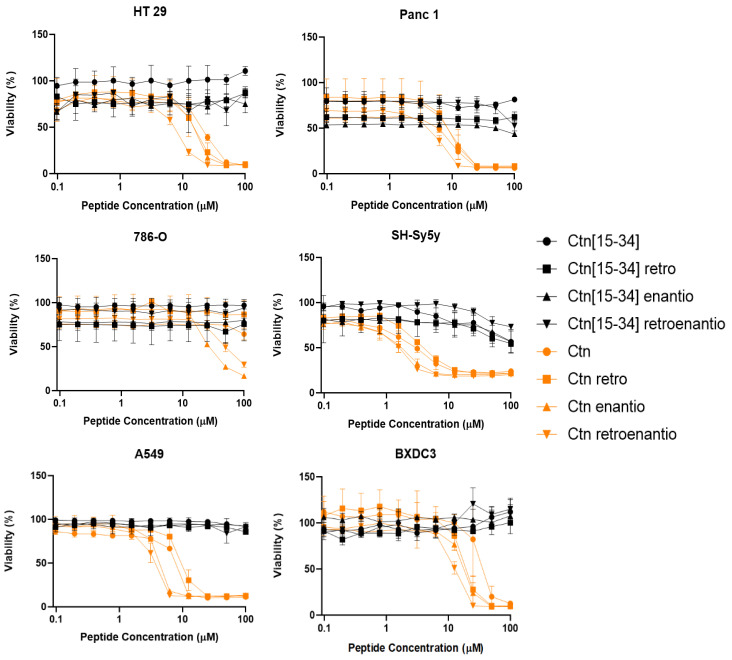
Viability of non-leukemia cells upon peptide treatment for 24 h. Ctn[15-34] and Ctn topoisomers are depicted in black and orange, respectively. Results are the average of three replicates.

The activity of Ctn, Ctn[15-34] and their topoisomers against U937, THP-1, Jurkat·6.1, HeLa S3, BxPC3, A549, SH-Sy5Y, Panc1, 786-O, and HT29 tumor cell lines was also studied. After incubation with peptide, cell viabilities were determined by resazurin, a nonfluorescent dye with a low metabolic activity that is reduced to fluorescent resorufin only by live cells and detected by a fluorescence spectrometer. As shown in Figure 1 and Figure 2, the peptides displayed selective toxicity in a concentration-dependent manner. Specifically, for leukemia cells (Figure 1), as previously reported [9], the most toxic peptide was Ctn, with IC_50_ values below 1 μM for Jurkat E6.1, THP-1, and U937; and in the low μM range (IC_50_ ≈ 3.47) for Hela S3. Similar results were also observed for the Ctn topoisomers. On the other hand, Ctn[15-34] and its topoisomers had little toxicity toward leukemia cells, with IC_50_ values in the mM range (Figure 1).

Similar toxicity studies were performed with other non-leukemia cell lines: BxPC3, A549, SH-Sy5Y, Panc1, 786-O, and HT29 (Figure 2). Ctn was again the most toxic peptide, particularly for neuroblastoma (SH-Sy5Y, IC_50_ ≈ 2.61 μM). The other cancer cell lines, with IC_50_ values near or above 10 μM, were not considered potential targets for either Ctn or its topoisomers. Likewise, Ctn[15-34] and its topoisomers had practically no effects on non-leukemia cells.

**Figure 3:**
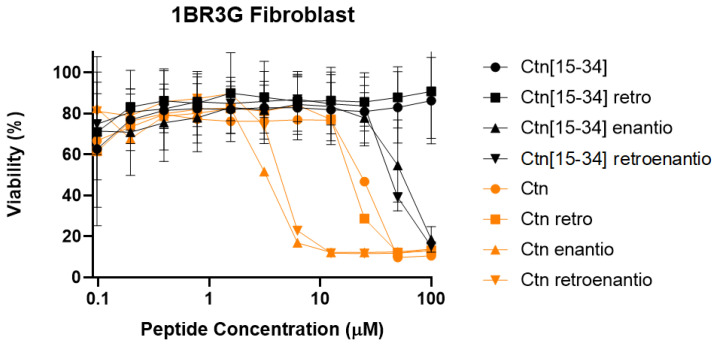
Toxicity of Ctn and Ctn[15-34] topoisomers to eukaryotic cells.

Cell viability of human fibroblast (1BR3G line derives from a normal fibroblast transformed with antigen T of SV40 (an oncogene), offering characteristics of a tumoral cell) after 24 h treatment with Ctn, Ctn[15-34], and their topoisomers was next investigated. Results showed that full-length Ctn and its topoisomers were more toxic than the Ctn[15-34] fragment and its congeners. Indeed, neither Ctn[15-34] (IC_50_ ≈ 14.32 μM) nor its retro version (IC_50_ ≈ 15.84 μM) were toxic. It is also worth noting the clear differences in terms of cell viability observed between D- and L-residue-containing peptides. Consistently, D-isomers were more toxic than the L- versions, with IC_50_ values of 2.7 μM and 4.3 μM for Ctn enantio and Ctn retroenantio, respectively (Figure 3), versus 191 μM and 43 μM for Ctn[15-34] enantio and Ctn[15-34] retroenantio.

### 3.4. Hemolytic Activity

**Figure 4:**
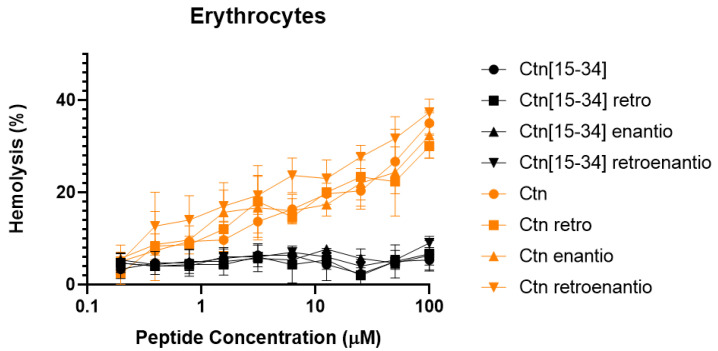
Hemolysis data of Ctn and Ctn[15-34] topoisomers.

The hemolytic effect of the peptides was tested on human erythrocytes and showed Ctn topoisomers as moderately lytic peptides (ca. 30% at 100 μM) (Figure 4). In contrast, Ctn[15-34] and all its topoisomers were totally nonhemolytic up to 100 μM.

### 3.5. Serum Stability

**Figure 5:**
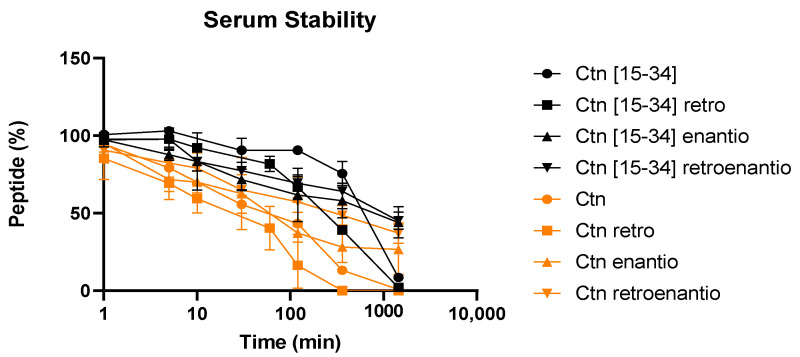
Stability of Ctn and Ctn[15-34] topoisomers in human serum.

All peptides were routinely tested for stability in human serum by LC-MS. Results confirmed the lower stability of Ctn and its topoisomers over Ctn[15-34]—previously reported as remarkably resilient to serum degradation [9,20]—and its counterparts. In general, the retro versions displayed stability trends similar to their cognates, although with slightly lower values (Figure 5). As expected, enantio and retroenantio peptides, by virtue of their D-amino acid content, were less susceptible to serum degradation, with 30% Ctn enantio and 40% Ctn retroenantio still remaining after 24 h exposure and up to 50% in the case of D-residue-containing Ctn[15-34] topoisomers. Of note was Ctn[15-34] retroenantio, with unusually high serum stability (t_1/2_ = 921 min), even for a linear enantiomeric peptide.

### 3.6. NMR Structures of Ctn and Ctn[15-34] Retroenantio Versions in DPC Micelles

**Figure 6:**
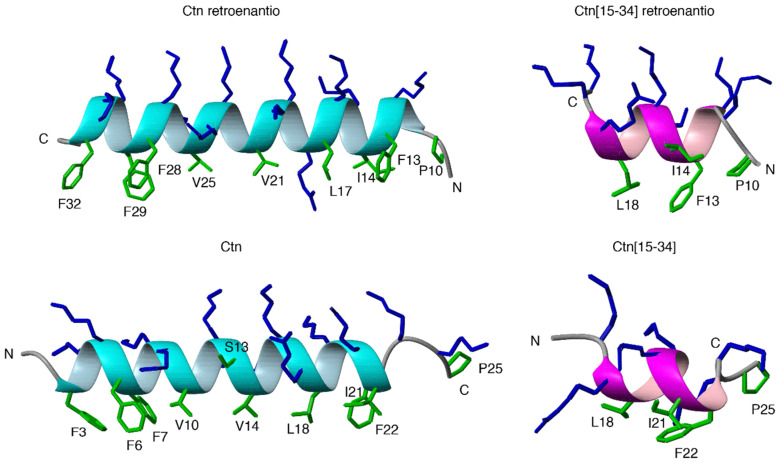
Ribbon representations of helical regions determined by NMR of Ctn retroenantio (**left**, **top**), Ctn (**left**, **bottom**), Ctn[15-34] retroenantio (**right**, **top**), and Ctn[15-34] (**right**, **bottom**) peptides. Arg and Lys side chains are shown in blue, and all other side chains are shown in green. N- and C-termini as well as hydrophobic side chains are labeled.

Structures of Ctn and Ctn[15-34] retroenantio peptides were characterized by solution NMR in a membrane-like (DPC micelle) environment and compared with Ctn and Ctn[15-34] original versions [9]. The two DPro residues present in the retroenantio peptides present *cis–trans* isomerism. The small differences in chemical shifts between the ^13^Cβ and ^13^Cγ for both Pro residues (δ^Pro^ = δ_Cβ_ − δ_Cγ,_ ppm; see Table S1) demonstrate that the rotameric state for both Pro in the two retroenantio peptides is *trans* [41], as was the case for the parent peptides [9]. Based on ^1^H and ^13^C chemical shift deviations (Figures S1 and S2), Ctn retroenantio forms a helix extending residues 11-33 (corresponding to residues 24-2 in parent Ctn), and Ctn[15-34] retroenantio a helix spanning residues 11-19 (corresponding to residues 24-16 in Ctn[15-34]). Thus, the helices formed by the retroenantio peptides are slightly longer than those in the cognates. In both peptides, the N-terminal segment (residues 1-10) is mainly disordered, similarly to the equivalent residues in the parent peptides. The helical populations are also similar between Ctn (79%) and Ctn retroenantio (78%) peptides, but helicity in Ctn[15-34] retroenantio peptide (56%) is higher than in the cognate fragment (15%) but lower than that of the entire Ctn retroenantio peptide (Table S2). To visualize the formed helices, structure calculations were performed using distance restraints derived from the experimentally observed NOEs (see Methods). The resulting structural ensembles show well-defined helices and a disorder N-terminal region (Figure S3 and Table S3). Figure 6 shows ribbon representations of these structures in comparison with those of the original versions. Despite the differences in helix lengths, the helices formed by the parent and retroenantio peptides displayed similar amphipathicity.

## 4. Discussion

Bioactive peptides isolated from natural sources, including animal toxins, plant extracts, fungi, and bacteria, have long raised considerable interest for their manifold therapeutic properties [42,43]. Among such peptides, snake-venom-derived cathelicidins are an interesting family with demonstrable promising anti-infective and antitumor activities. Our group has contributed to this field by showing that crotalicidin (Ctn) and Ctn[15-34], a selected fragment thereof, kill bacteria through disruption of their membrane following three stages [19]: (1) initial peptide recruitment by electrostatic interactions, (2) surface accumulation, and (3) bacterial death by membrane disruption. Ctn[15-34] is additionally remarkable for its long-term stability in human serum [20] and low toxicity in healthy eukaryotic cells [9]. Even so, as natural peptides composed of L-amino acids, both Ctn and Ctn[15-34] remain susceptible to proteases and thus amenable to improvement by peptide engineering methods.

The enantiomers (D-amino acid versions) of natural bioactive peptides have been explored as a means to overcome the protease susceptibility of the native forms [20]. Unfortunately, as only a small fraction of biologically relevant interactions are chirality-indifferent, the application of strict enantiomers of bioactive peptides is confined to a few specific areas such as AMPs or cell-penetrating peptides (CPPs) [44,45]. In contrast, the retroenantio (*re*) approach [23], where the original bioactive sequence is reversed and made up of D-amino acids hence highly protease-resistant, has over recent years shown multiple examples of therapeutic promise. For instance, THR*re*, the retroenantio version of THR (THR is a peptide that interacts with the transferrin receptor but does not compete with transferrin [46,47]), has better blood–brain barrier (BBB)-crossing properties than its cognate, extensively degraded by proteases [24]. Our own group, applying the *re* approach, has recently developed an orally active BBB-crossing peptide candidate that minimizes the adverse cognitive effects of cannabinoid therapy while preserving analgesic properties [25].

In tune with these approaches, in this work, we have explored the structural and functional properties of the topoisomers of rattlesnake-derived AMPs crotalicidin (Ctn) and Ctn[15-34]. MIC assays showed the topoisomers to be effective AMPs (Table 2), particularly against Gram-negative bacteria, as previously reported for the cognate Ctn and Ctn[15-34] [9]. In addition, antitumoral screens [12,48] on various leukemia and HeLa cell lines showed Ctn topoisomers to be more cytotoxic than Ctn[15-34] analogs, also in agreement with previous observations [9]. Toxicity assays were extended to other cancer cell lines, showing an IC_50_ of 2.61 μM for Ctn against neuroblastoma that merits further study.

While the enantio and retroenantio versions, in general, confirmed expectations of enhanced serum survival (Section 3.5, Figure 5), they were, unfortunately, slightly more toxic in eukaryotic cells than the original L-peptides. The most probable reason for this toxicity is that mammalian cells, unlike bacteria, do not have racemases directly converting D- into L-amino acid residues [49]; an alternative two-step conversion pathway has been postulated, i.e., (i) transformation to α-keto acids by D-amino acid oxidase and D-aspartate oxidase; (ii) transamination to L-amino acids [50]. Unfortunately, this sequence produces H_2_O_2_ as a by-product, accumulation of which may result in lipid peroxidation and cell membrane damage [51].

Finally, solution NMR studies of Ctn and Ctn[15-34] retroenantio versions showed an increase in helix length in both peptides compared to their corresponding L-precursors, with a significant increase in the helix population of Ctn[15-34] retroenantio. Taking into account the well-known principles of helix stability [35], these differences could be explained by the fact that stabilizing contributions in α-helices are position-dependent, with the N-cap and C-cap residues playing an important role. For instance, because of the helix dipole, positively charged Lys and Arg favor helix formation at the C-terminal region and are helix-destabilizing at the N-terminal region. In our case, R18 and K19 located at the C-terminus of Ctn[15-34] retroenantio are very likely contributing to increase helix population, whereas the equivalent residues (R3 and K2) in the parent Ctn[15-34] peptide are likely to play an opposite role and decrease helix population at the N-terminus.

These structural issues notwithstanding, no obvious relation between helix population and activity can be inferred. The mechanism of action of Ctn[15-34] and Ctn in disrupting membranes is by electrostatic interaction between the positively charged peptide and the anionic head groups of bacterial membrane phospholipids [19]. The activities of the different Ctn and Ctn[15-34] topoisomers against Gram-negative bacteria are quite similar, suggesting that the interaction is predominantly electrostatic; hence, peptide 3D structure is of marginal significance. Both of the topoisomers studied preserve an amphipathic helix structure with a hydrophobic face that may interact with membrane lipids and a hydrophilic side compatible with aqueous environments [52].

This study was launched in the hope that Ctn[15-34], a peptide long-studied in our group for its promising combination of antimicrobial and antitumor properties with singular serum stability, could be further improved therapeutically by way of retro or retroenantio congeners. Unfortunately, such expectations were not obtained, and Ctn[15-34] in its original all-L version remains more competitive than any topoisomer. In contrast, Ctn retroenantio is more promising than Ctn and, therefore, worth investigating alongside Ctn[15-34] in in vivo studies.

## Figures and Tables

**Table 1:** Primary structures of crotalicidin, Ctn[15-34] and their topoisomers.

Peptides	Sequence ^1^	Charge ^2^	Hydrophobicity ^3^
Crotalicidin (Ctn)	KRFKKFFKKVKKSVKKRLKKIFKKPMVIGVTIPF	+16	0.263
Ctn retro	FPITVGIVMPKKFIKKLRKKVSKKVKKFFKKFRK	+16	0.263
Ctn enantio	krfkkffkkvkksvkkrlkkifkkpmviGvtipf	+16	0.263
Ctn retroenantio	fpitvGivmpkkfikklrkkvskkvkkffkkfrk	+16	0.263
Ctn[15-34]	KKRLKKIFKKPMVIGVTIPF	+8	0.455
Ctn[15-34] retro	FPITVGIVMPKKFIKKLRKK	+8	0.455
Ctn[15-34] enantio	kkrlkkifkkpmviGvtipf	+8	0.455
Ctn[15-34] retroenantio	fpitvGivmpkkfikklrkk	+8	0.455

^1^ All peptides are C-terminally amidated. ^2^ At neutral pH, from http://pepcalc.com/ (accessed on 18 August 2022). ^3^ From https://heliquest.ipmc.cnrs.fr/cgi-bin/ComputParams.py (accessed on 18 August 2022).

**Table 2:** Minimal inhibitory concentrations (MICs) of Ctn, Ctn[15-34], and their topoisomers against Gram-negative bacteria.

	MIC (μM)		
Peptide	*E. coli*	*A. baumannii*	*Pseudomonas* sp.
Ctn	<0.05	0.78	<0.05
Ctn enantio	0.39	1.56	0.39
Ctn retro	0.78	1.56	1.56
Ctn retroenantio	0.78	1.56	0.78
Ctn[15-34]	<0.005	0.78	<0.05
Ctn[15-34] enantio	0.16	0.39	0.1
Ctn[15-34] retro	0.16	0.78	<0.05
Ctn[15-34] retroenantio	0.16	0.39	0.1

## Data Availability

NMR assigned chemical shifts have been deposited at the BioMagResBank (http://www.bmrb.wisc.edu (accessed on 28 July 2022)) with accession codes 51536 (Ctn retroenantio) and 52537 (Ctn[15-34] retroenantio).

## Supplementary Materials

The following are available online at https://www.mdpi.com/article/10.3390/biomedicines10092110/s1: NMR data (Tables S1–S3; Figures S1–S3); analytical data of peptides (Table S4); HPLC traces and LC-MS spectra of peptides.
