# Supplementary material for: Examining Topoisomers of a Snake-Venom-Derived Peptide for Improved Antimicrobial and Antitumoral Properties
Source: Biomedicines. 2022 Aug 29;10(9):2110. doi: 10.3390/biomedicines10092110 (PMC9495681; doi:10.3390/biomedicines10092110)

Supplementary information

Examining Topoisomers of a Snake-Venom-Derived Peptide for Improved Antimicrobial and Antitumoral Properties

Adam Carrera-Aubesart [1], Sira Defaus [1,*], Clara Pérez-Peinado [1], Daniel Sandín [2], Marc Torrent [2], Maria Ángeles Jiménez [3] and David Andreu [1,*]

[1] Proteomics and Protein Chemistry Unit, Department of Medicine and Life Sciences, Pompeu Fabra University, 08003 Barcelona, Spain
[2] Systems Biology of Infection Lab, Department of Biochemistry and Molecular Biology, Facultat de Biociències, Universitat Autònoma de Barcelona, 08193 Bellaterra, Spain
[3] Institute of Physical Chemistry "Rocasolano" (IQFR), Consejo Superior de Investigaciones Científicas (CSIC), 28006 Madrid, Spain
* Correspondence: sira.defaus@upf.edu (S.D.); david.andreu@upf.edu (D.A.)

Contents

	Pages
**1. NMR**	
Table S1	S4
Table S2	S5
Table S3	S6
Figure S1	S7
Figure S2	S8
Figure S3	S9
**2. Analytical data**	
Table S4	S11
HPLC-MS analytical data	S12-S14

1. NMR

Table S1. Differences in chemical shifts between the $^{13}C_{\beta}$ and $^{13}C_{\gamma}$ ($\Delta\delta^{Pro} = \delta_{C\beta} - \delta_{C\gamma}$, ppm) for the D-Pro residues of the retroenantio Ctn and Ctn[15-34] peptides 30 mM DPC, pH 3.0, at 35°C.

Peptide	Residue	$\delta_{C\beta}$, ppm	$\delta_{C\gamma}$, ppm	$\Delta\delta^{Pro}$, ppm
Ctn retroenantio	D-Pro 2	32.0	27.4	4.6
	D-Pro 10	31.3	27.9	3.4
Ctn[15-34] retroenantio	D-Pro 2	32.0	27.4	4.6
	D-Pro 10	31.6	27.8	3.8

Table S2. Averaged $\Delta\delta_{H\alpha}$ values ($\Delta\delta_{H\alpha} = \delta_{H\alpha}^{observed} - \delta_{H\alpha}^{RC}$, ppm) and % helix estimated from these values for the parent and retroenantio Ctn and Ctn[15-34] peptides in 30 mM DPC, pH 3.0, at 35°C. $\delta_{H\alpha}^{RC}$ values were taken from Wishart et al. 1995. The averaged $\Delta\delta_{H\alpha}$ values and the % helix for the parent Ctn and Ctn[15-34] peptides were calculated from chemical shifts deposited at BioMagResBank with accession codes 25363 and 25370, respectively. [a] Values in parenthesis indicate the corresponding residues in the parent peptides.

Peptide	Helical length	Helical residues	$\Delta\delta_{H\alpha}$, ppm	% helix
Ctn retroenantio	23	11-33 (24-2)[a]	-0.31	78
Ctn[15-34] retroenantio	9	11-19 (24-16)[a]	-0.22	56
Ctn	19	3-21	-0.31	79
Ctn[15-34]	6	18-23	-0.06	15

Table S3. Statistical structural parameters for the ensemble of the 20 lowest target function conformers calculated for retroenantio Ctn and Ctn[15-34] peptides in DPC-micelles. [a]Residues taken into consideration to calculate RMSD are indicated between brackets.

	Ctn retroentantio	Ctn[15-34] retroenantio
**Number of distance restraints**		
Intraresidue (|i – j| = 0)	102	108
Sequential (|i – j| = 1)	72	83
Medium range (1 < |i – j| < 5)	83	74
Total number	257	265
Averaged total number per residue	7.6	13.3
**Number of H-bond restraints**	18	5
**Averaged maximum violations per structure**		
Distance (Å)	0.07 ± 0.01	0.08 ± 0.05
**Averaged CYANA target function value**	0.03 ± 0.01	0.11 ± 0.03
**Pairwise RMSD (Å)[a]**		
All atoms		
Backbone atoms	3.8 ± 1-3 (2-33)	2.6 ± 1.4 (2-19)
All heavy atoms	0.3 ± 1.2.1 (11-32)	3.4 ± 1.3 (2-19)
Helical residues		
Backbone atoms	4.4 ± 0.2 (3-21)	0.13 ± 0.06 (12-18)
All heavy atoms	1.6 ± 0.2 (11-32)	1.0 ± 0.3 (12-18)

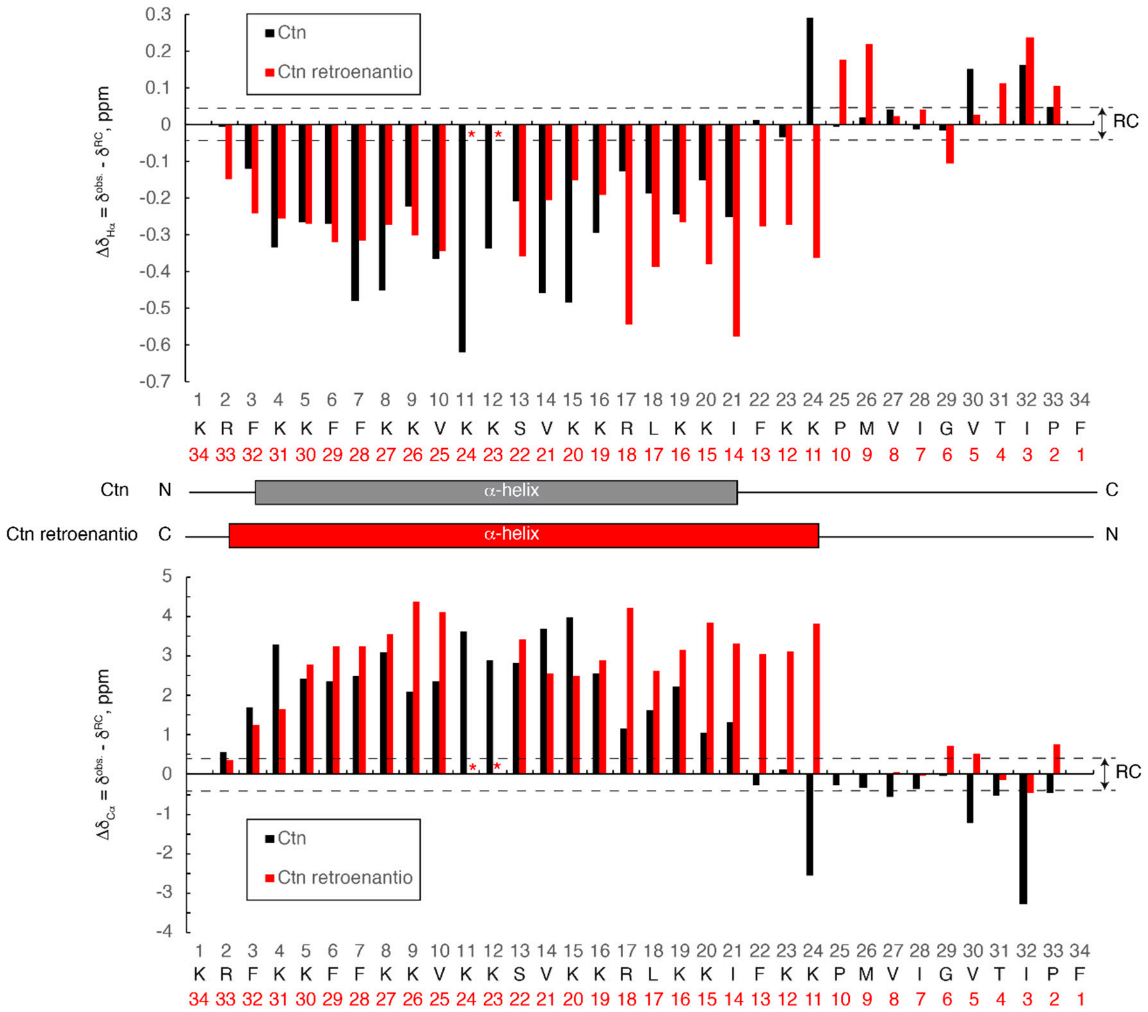

Figure S1. Bar plots of $\Delta\delta_{H\alpha}$ ($\Delta\delta_{H\alpha} = \delta_{H\alpha}{}^{observed} - \delta_{H\alpha}{}^{RC}$, ppm) and $\Delta\delta_{C\alpha}$ ($\Delta\delta_{C\alpha} = \delta_{C\alpha}{}^{observed} - \delta_{C\alpha}{}^{RC}$, ppm) as a function of sequence for the Ctn (black bars) and the retroenantio Ctn-RE (red bars) in DPC micelles (30 mM DPC) at pH 3.0 at 35°C. Values for N- and C-terminal residues are not displayed. $\delta_{H\alpha}{}^{RC}$ and $\delta_{C\alpha}{}^{RC}$ were taken from Wishart et al. 1995. $\delta_{H\alpha}{}^{observed}$ and $\delta_{C\alpha}{}^{observed}$ values for the parent Ctn peptide were taken from chemical shifts deposited at BioMagResBank with accession code 25363. Values for N-and C-terminal residues are not shown. Note that to compare equivalent residues the sequence of the retroenantiomer is inverted in the plot. Residue numbers at the x-axis are in black for Ctn and in red for retroenantio Ctn. Asterisks indicate values which could not be determined. Horizontal dashed lines indicate the random coil (RC) range.

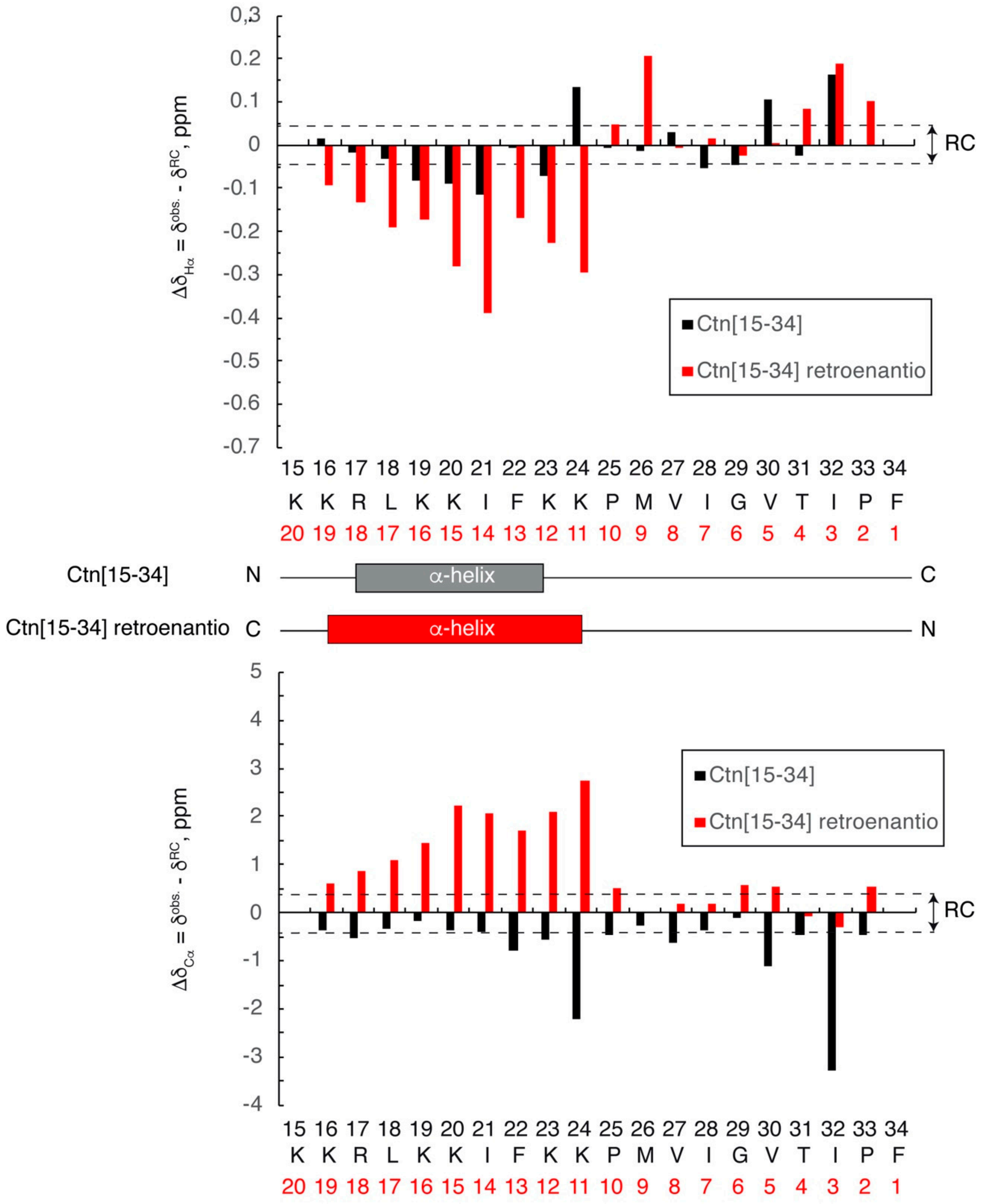

Figure S2. Bar plots of $\Delta\delta_{H\alpha}$ ($\Delta\delta_{H\alpha} = \delta_{H\alpha}^{observed} - \delta_{H\alpha}^{RC}$, ppm) as a function of sequence for Ctn[15-34] (black; sequence from C-to-N-end) and Ctn[15-34]-RE (red; sequence from N-to C-end) in aqueous solution and in DPC micelles (30 mM DPC) at pH 3.0 and 35°C. $\delta_{H\alpha}^{RC}$ and $\delta_{C\alpha}^{RC}$ were taken from Wishart et al. 1995. Values for N-and C-terminal residues are not shown. $\delta_{H\alpha}^{observed}$ and $\delta_{C\alpha}^{observed}$ values for the parent Ctn[15-34] peptide were taken from chemical shifts deposited at BioMagResBank with accession code 25370. Note that to compare equivalent residues the sequence of the retroenantiomer is inverted in the plot. Residue numbers at the x-axis are in black for Ctn and in red for Ctn retroenantio. Asterisks indicate values which could not be determined. Horizontal dashed lines indicate the random coil (RC) range.

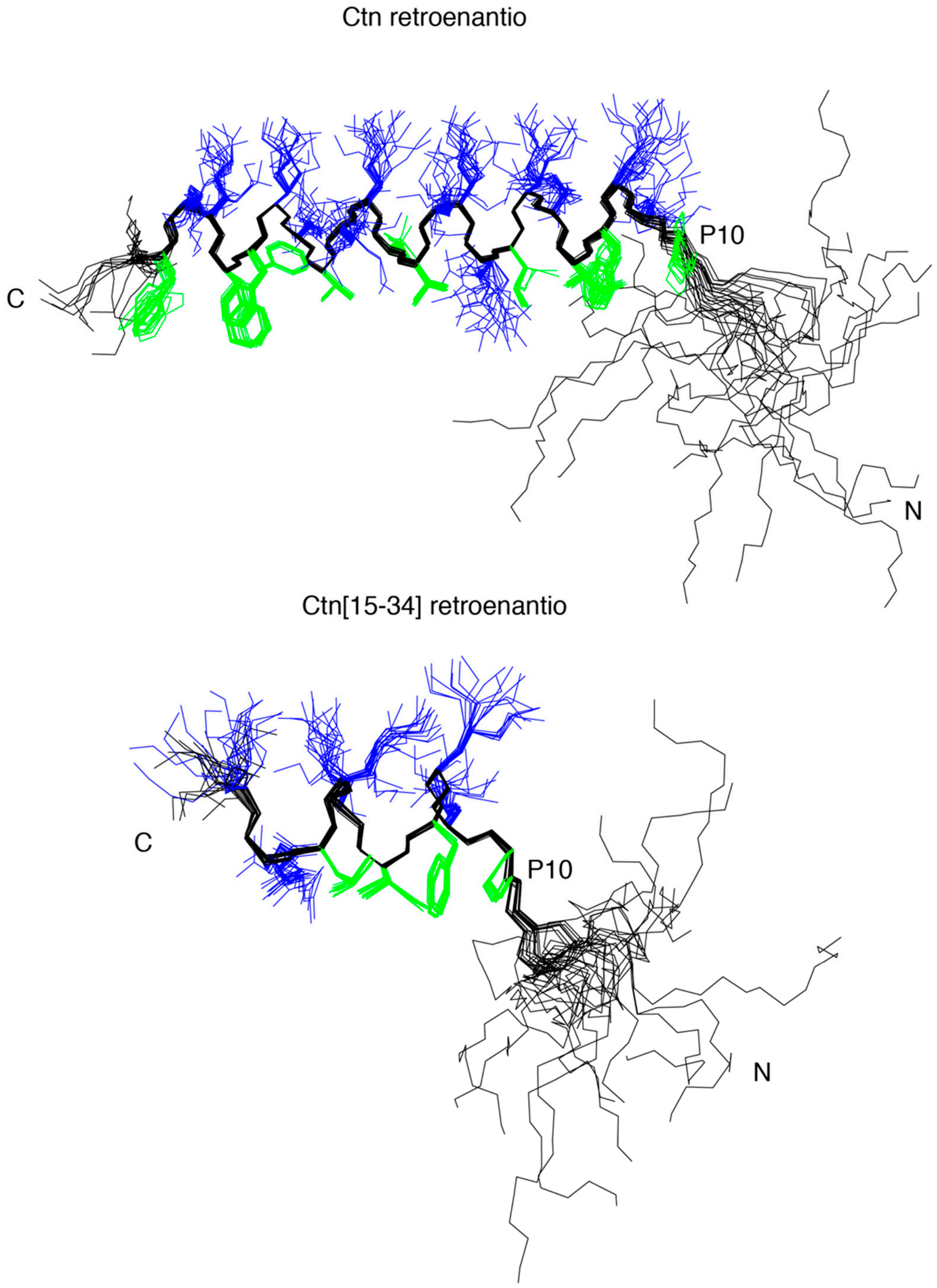

Figure S3. NMR structure of Ctn retroenantio (top) and Ctn[15-34] retroenantio (bottom) in DPC micelles. Overlay of the 20 lowest target function conformers. Backbone atoms are in black, Arg and Lys sidechains are in blue, and all other sidechains in green. N and C-termini, as well as Pro 10 are labelled.

2. Analytical data

Table S4. Peptides analytical data.

Peptides[1]	Theoretical mass (Da)	Experimental mass (Da)[2]	HPLC retention time (min)[3]	Purity[4]
Crotalicidin (Ctn)	4151.36	4151.40	7.0	97%
Ctn retro	4151.36	4151.40	8.0	94%
Ctn enantio	4151.36	4151.00	7.0	98%
Ctn retroenantio	4151.36	4151.00	8.1	97%
Ctn [15-34]	2371.08	2370.40	6.5	99%
Ctn [15-34] retro	2371.08	2370.00	6.6	99%
Ctn [15-34] enantio	2371.08	2370.80	6.6	98%
Ctn [15-34] retroenantio	2371.08	2370.60	6.6	97%

[1] All peptides are C-terminal carboxamides; [2] Determined by LC-MS; [3] Elution was carried out with a 10 to 50 linear gradient over 15 min; [4]Determined by analytical HPLC.

HPLC-MS analysis. Left: HPLC traces of purified peptide products. Right: ESI-MS spectra of pure peptides.

Ctn: KRFKKFFKKVKKSVKKRLKKIFKKPMVIGVTIPF

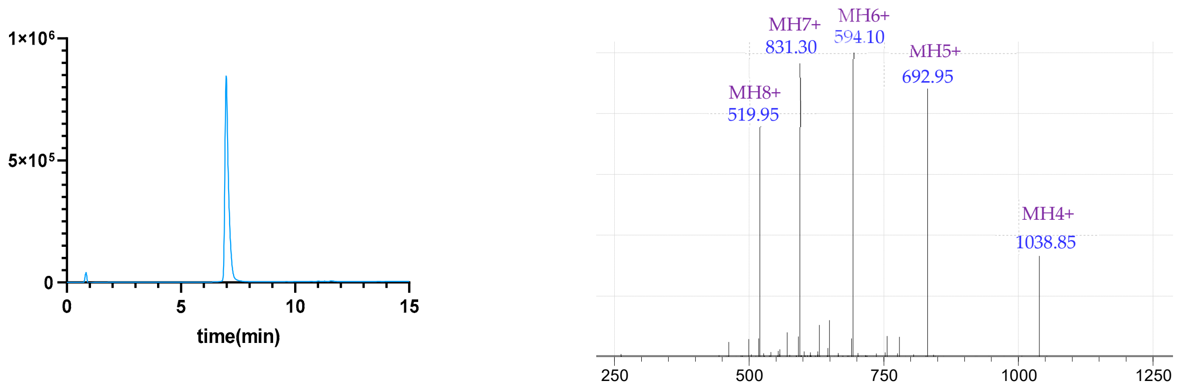

Ctn retro: FPITVGIVMPKKFIKKLRKKVSKKVKKFFKKFRK

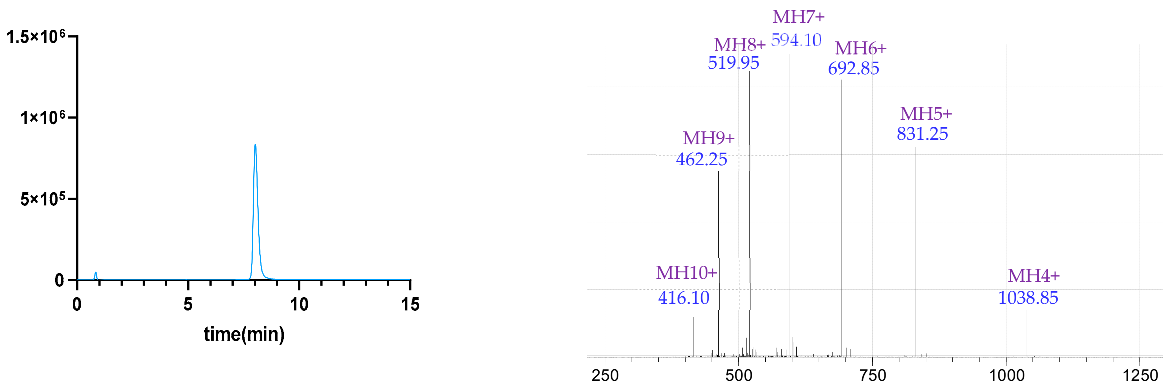

Ctn enantio: krfkkffkkvkksvkkrlkkifkkpmviGvtipf

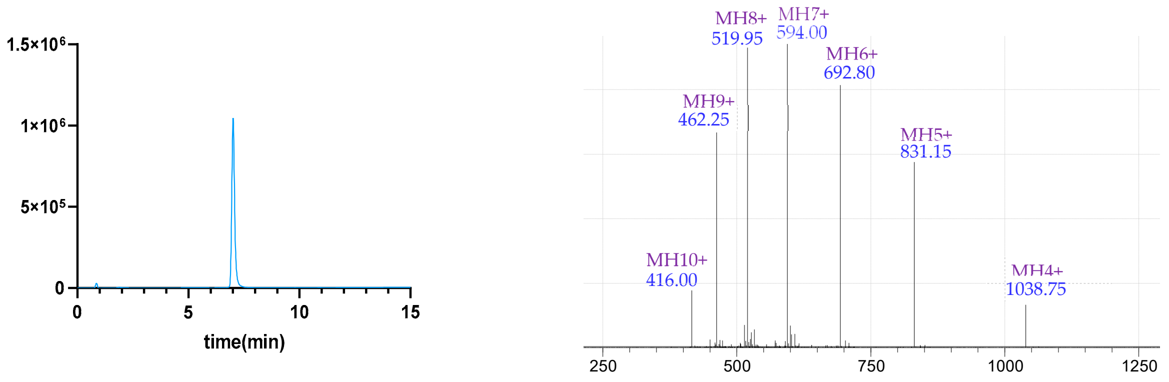

Ctn retroenantio: fpitvGivmpkkfikklrkkvskkvkkffkkfrk

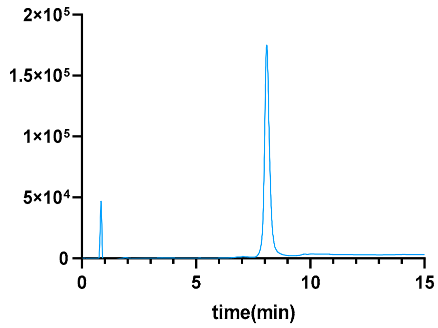

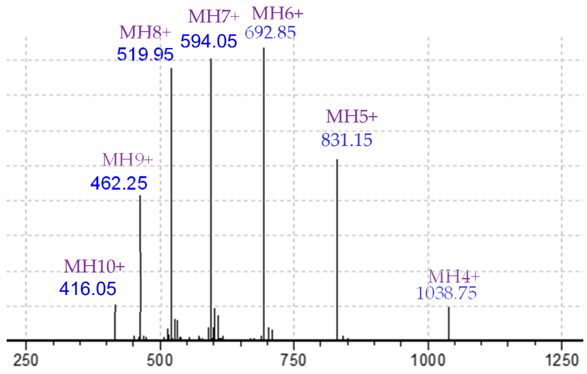

Ctn[15-34]: KKRLKKIFKKPMVIGVTIPF

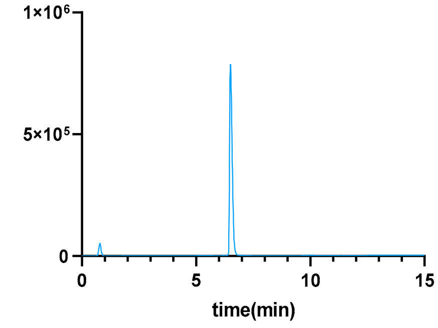

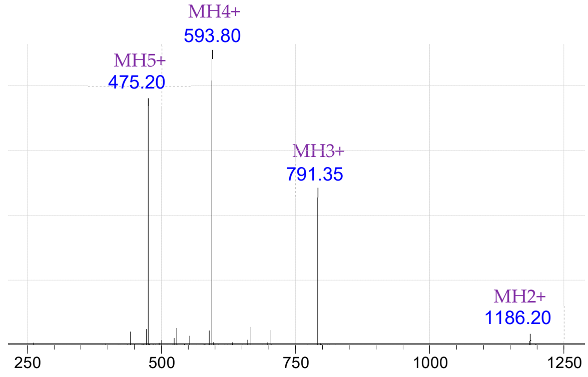

Ctn[15-34] retro: FPITVGIVMPKKFIKKLRKK

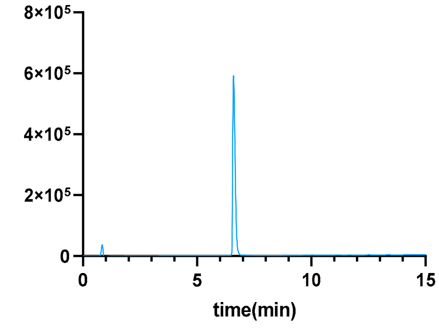

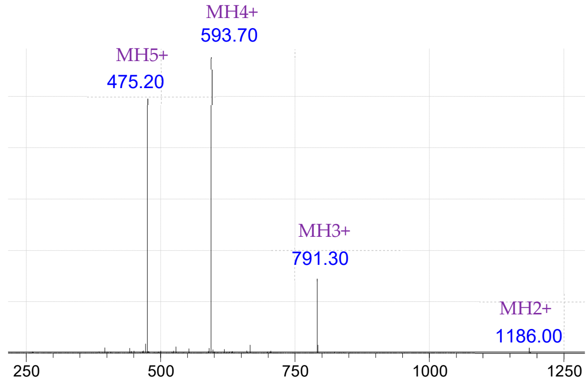

Ctn[15-34] enantio: kkrlkkifkkpmviGvtipf

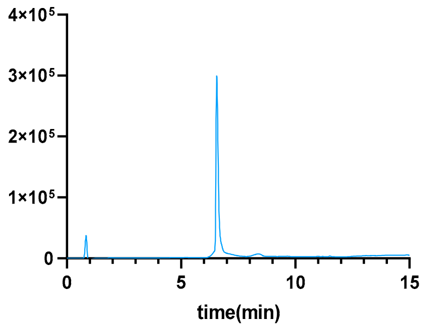

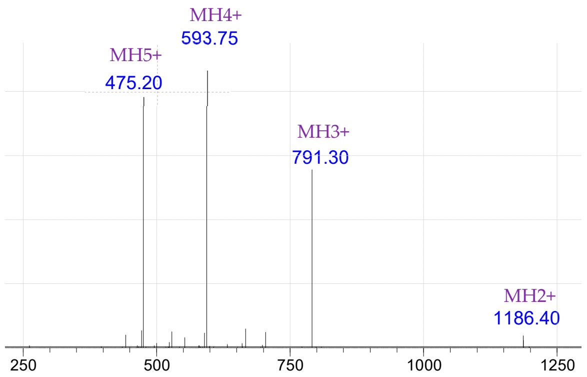

Ctn[15-34] retroenantio: fpitvGivmpkkfikklrkk

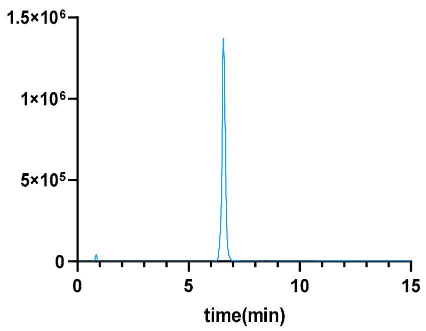

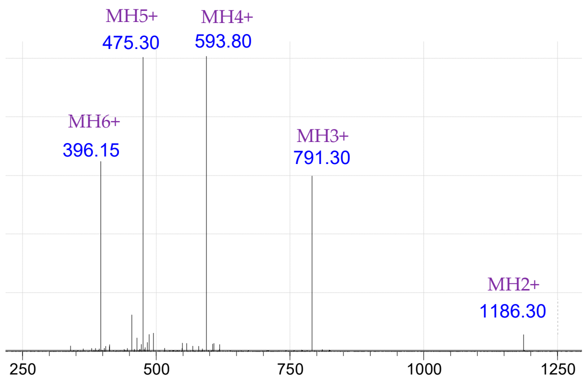

Supplement: Supplementary file 1 [file biomedicines-10-02110-s001.zip › biomedicines-1865599-supplementary.pdf]
